# Alteration of Blood Oxidative Stress Status in Patients with Thoracic Aortic Dissection: A Pilot Study

**DOI:** 10.3390/antiox12051106

**Published:** 2023-05-16

**Authors:** Joël Pincemail, Vincent Tchana-Sato, Audrey Courtois, Lucia Musumeci, Jean-Paul Cheramy-Bien, Jacobine Munten, Nicos Labropoulos, Jean-Olivier Defraigne, Natzi Sakalihasan

**Affiliations:** 1 Department of Cardiovascular Surgery, CHU Liege, 4000 Liège, Belgium; 2 Department of Medical Chemistry, CHU Liege, 4000 Liège, Belgium; 3 CHC Montlegia, Onco-Hematology, 4000 Liège, Belgium; 4 Department of Surgery, Stony Brook University Hospital, Stony Brook, NY 11794-8191, USA

**Keywords:** human thoracic aortic dissection, oxidative stress, inflammation, antioxidants, blood biomarkers

## Abstract

Background: Thoracic aortic dissection (TAD) is a life-threatening condition which usually occurs on an aneurysmal aortic wall. Although increasing data have shown that inflammation and oxidative stress play an important role in the patho-physiology of dissection, systemic oxidative stress status (OSS) has not been clearly determined in patients suffering from TAD. Methods: A cohort of 115 patients presenting type A or B TAD were admitted to our center from 2013 to 2017. Out of this cohort, 46 patients were included in a study on dissected aorta (LIege study on DIssected Aorta: LIDIA). In 18 out of the 46 patients, systemic OSS parameters were evaluated after TAD diagnosis by determination of eight different antioxidants, four trace elements, two markers of oxidative lipid damage and two inflammatory markers. Results: The 18 TAD patients included 10 men and 8 women (median age: 62 years; interquartile range: 55–68) diagnosed with type A (N = 8) or B (N = 10) TAD. Low plasma levels of vitamin C, β-carotene, γ-tocopherol, thiol proteins, paraoxonase and selenium were observed in these 18 patients. By contrast, the concentration of copper and total hydroperoxides, copper/zinc ratio, as well as inflammatory markers, were higher than the reference intervals. No difference was observed in oxidative stress biomarker concentrations between type A and B TAD patients. Conclusions: This pilot study, limited to 18 TAD patients, revealed a heightened systemic OSS, determined at 15.5 days (median) after the initial diagnosis, in those TAD patients without complications (malperfusion syndrome and aneurysm formation). Larger studies on biological fluids are needed to better characterize the oxidative stress and interpret its consequence in TAD disease.

## 1. Introduction

Thoracic aortic dissection (TAD) is a serious and potentially life-threatening condition characterized by the presence of an “intimal flap” which results from the separation of the true lumen from the false one [1]. Depending on the location of the tear within the aorta, dissections are classified as type A, when the tear occurs at the ascending aorta, or type B, whenever the tear is in the descending aorta, according to the Stanford classification [2]. The progression of blood flow within the aortic wall leads to an anterograde and retrograde extension of the intimal flap which can lead into static or dynamic malperfusion of some organs, acute aortic valve defect and tamponade. Population-based studies suggest the incidence of TAD to be around 35 cases per 100,000 people per year in the population aged 65 to 75 years [3]. Despite substantial improvement in the diagnosis and the management of acute aortic diseases, the underlying pathological mechanism leading to TAD has not been fully elucidated.

Whether due to the inherent instability of the aortic wall or an acquired condition, compromised aortic integrity is a fundamental component of the underlying dissection pathology [4]. Increased inflammation processes, contributing to extracellular matrix destruction as evidenced by accumulation of macrophages and neutrophils [5], appears to be an important molecular mechanism involved in the pathogenesis of aortic dissection [6]. Upon activation, neutrophils release proteases (elastase) and metalloproteases (MMP9) into the extracellular matrix that contribute to wall degradation [7].

An increasing number of studies have highlighted the key role of oxidative stress (OS) in the progression of cardiovascular diseases including abdominal aortic aneurysm (AAA) [8] and TAD [9,10].

OS has been defined as an imbalance between reactive oxygen species (ROS) and antioxidants in favor of the former, leading to a disruption of signaling as a consequence of irreversible oxidative damage to lipids, DNA or proteins [11]. This definition takes into account both the physiological effects of ROS in the regulation of cellular homeostasis and their pathological incidence in the development of human pathologies.

As recently reviewed, the main enzymatic sources of increased ROS production in TAD are mitochondrial and endothelial dysfunctions, xanthine oxidase, myeloperoxidase, NADPH oxidase and the endoplasmic reticulum [12,13]. In order to regulate ROS production and combat their deleterious effect, the organism has a vast network of antioxidants [14] including enzymes (superoxide dismutase, catalase, glutathione peroxidase, etc.) and low-molecular-weight molecules (vitamins C and E, glutathione, ubiquinone, carotenoids, polyphenols), with the latter being provided by the diet.

Some studies have evidenced the presence of increased OS in the aortic segments of patients with congenital bicuspid aortic valve, and Loeys–Dietz and Marfan syndromes, which all contribute to the development of TAD [15,16,17,18,19,20]. Such observations in dissected thoracic aortic segments are based on an accumulation of superoxide anions and lipid peroxidation products as well as a reduced amounts of both enzymic and non-enzymic antioxidants.

Besides data on aortic tissues, only scarce information is available about an alteration in the systemic oxidative stress status (OSS) in TAD patients and it involves only a small number of OS biomarkers [21,22]. By contrast, there is no information about the OSS of TAD patients a few days after their diagnosis.

In order to fill in this gap, the present study has the goal to determine the presence of OS in the systemic circulation of TAD patients using a large battery of tests (N = 16) available in routine practice. This includes the analysis of both enzymic and non-enzymic antioxidants, trace elements, markers of oxidative damage to lipids and finally inflammation biomarkers.

In the past we have used such analyses to highlight an increased OS in patients with AAA, COVID-19, chronic obstructive pulmonary disease and facioscapulohumeral dystrophy [8,23,24,25,26].

## 2. Materials and Methods

### 2.1. Patients and Study Design

In an attempt to predict morphological and functional evolution of TAD, 115 patients diagnosed with type A or type B TAD who were admitted to our center from 2013 to 2017 were invited to participate in a study on dissected aorta in Liege (Liege study on dissected aorta, LIDIA). The exclusion criteria of the LIDIA study were aneurysm development; complications due to malperfusion, such as stroke, gastrointestinal ischemia, acute kidney insufficiency, liver insufficiency, acute limb ischemia and hemodynamic instability, making the patients unable to sign the informed consent form; or patient refused to participate. Out of the 115 TAD patients, 46 were included in the LIDIA study. This prospective study combined functional positron emission tomography (PET) computed tomography (CT) imaging of 18-fluorodeoxyglucose (18F-FDG) uptake and measurements of circulating biomarkers of coagulation/fibrinolysis. In addition, genetic analyses were also performed on patients’ blood genomic DNA in order to identify potential connective tissue disorders associated with TAD. Throughout the LIDIA study, therapeutic decisions were based on current clinical and morphological guidelines. In particular, for type A TAD, open ascending aorta surgery was performed in all cases, and for type B TAD a medical treatment (beta-blockers, antihypertension, pain killers) and/or thoracic endovascular aortic repair (TEVAR) was performed. Patient characteristics such as age, height, weight, arterial blood pressure, smoking habits, past medical history, and medications were collected the day of the admission.

During the course of the LIDIA study, we received a special grant for evaluating the systemic OSS of the last eighteen consecutive patients (8 with type A and 10 with type B dissection) 15.5 days (median value, IQR: 11.5–25.75 days) after the initial diagnosis of TAD, leading either to an emergency operation or a medical treatment. The study protocol was approved by the University Hospital Ethics Committee for Medical Research (2014/175, Nr Eudra CT 2014-002614-23). All the participants were instructed on the study objectives and signed an informed consent.

### 2.2. Blood Sample for OSS Analysis

The day before sampling, subjects were asked to fast for at least 12 h and not to drink fruit juice. Between 8:00 and 9:00 a.m., blood samples were drawn from the antecubital vein in tubes containing EDTA or Na-heparin as the anticoagulant or clot activating gel according to the investigated parameter. Blood samples were immediately centrifuged on site. Plasma or sera were then frozen as aliquots at −80 °C until analysis, which was performed within four days after blood collection.

### 2.3. Determination of OS Biomarkers

Analysis protocols for antioxidants (vitamins C and E, thiol proteins (PSH), β-carotene, glutathione peroxidase (GPx)), trace elements (selenium (Se), copper (Cu), zinc (Zn)), biomarkers of lipid peroxidation (total hydroperoxides (tROOH), oxidized LDL (ox-LDL)) have been previously described in detail by our group [8,23,24,25,26]. Plasma paraoxonase (PON) activity was determined by spectrophotometry using the hydrolysis of paraoxon as described earlier [27]. Interleukin 6 (IL-6) was detected in plasma EDTA using a High Sensitivity Human IL-6 Quantikine Elisa kit purchased from R&D, Abingdon, UK. C-reactive protein (CRP) concentration was determined using a COBAS^®^8000 analyzer (Roche Diagnostics, Machelen, Belgium).

All OS analyses were performed in a routine clinical setting at the Laboratory of Medical Chemistry of the University Hospital of Liège (CHU) according to the International Federation of Clinical Chemistry and Laboratory Medicine (IFCC) guidelines. All analyses were ISO certified (ISO15189).

### 2.4. Reference Population

In medical chemistry laboratories, a minimum of 100 healthy subjects is required to validate the analytical performance of methods (some of the parameters that are determined include precision, linearity, carry-over and reference intervals) in agreement with the CLSI EP23A3c guidelines. In the particular case of OS biomarkers, analyzed in the clinical routine in our center, we established our own reference intervals (regularly updated) using the ELAN (Etude Liègeoise sur les Antioxydants) cohort of 897 healthy subjects (349 men and 548 women aged 40–60 years) [28] following such guidelines. Furthermore, we performed another small study on 18 healthy older subjects (>65 years) and showed that their OS biomarkers were within our previously established reference intervals [29]. We have already used such reference intervals in previous studies on patients with AAA [8] and in two recent studies on COVID-19 patients [23,24]. Concentrations of each OS biomarker in TAD patients were compared to the concentrations obtained in this reference population (see statistical analysis).

### 2.5. Statistical Analysis

Quantitative data were expressed as median and interquartile range (IQR). The Spearman correlation coefficient was calculated to measure the association between biological parameters. The distribution of each biological parameter of the TAD cohort (N = 18) was compared to the laboratory reference interval from healthy individuals, using the sign test based on the binomial distribution [30,31]. Based on this test, in the case where all the biomarker values of the 18 TAD patients fall below or above the middle of the reference interval for the same biomarker, it can be concluded that the difference in concentration between TAD patients and healthy subjects is statistically relevant with a *p*-value < 0.0001. In the case where 17 of the 18 TAD patients (17/18) are below or above the middle of the reference interval, the probability is <0.001. The probabilities are *p* < 0.002 for 16/18 patients and *p* = 0.008 for 15/18 patients. The results are considered significant at the 5% critical level (*p* < 0.05).

Correlation between biological factors was established using the Spearman test for nonparametric values. A *p*-value < 0.05 was considered significant. Mann–Whitney U test for non-parametric values or the Wilcoxon signed-rank test were used to compare the median values between smokers and hypertensive patients.

## 3. Results

### 3.1. Characteristics and Risk Factors of the TAD Cohort

The present study included 10 men and 8 women (median age: 62 years; IQR: 55–68) diagnosed with Stanford type A (N = 8) or B (N = 10) TAD (Table 1). Only four patients (22%) never smoked and the majority had hypertension (78%). All the patients with type A TAD underwent urgent open surgery (supracoronary ascending aorta replacement (N = 4); supracorononary ascending aorta replacement + coronary artery bypass (N = 1); root replacement Bentall (N = 3)). Among the patients with type B TAD, eight were treated medically, while two required TEVAR. There was no in-hospital death in our cohort.

### 3.2. OS Biomarkers in the TAD Cohort

For each OS parameter, Table 2 displays the reference intervals, the median (IQR) for all 18 TAD patients, the number of blood values (K) below (or above) the middle of the reference interval. Using the sign test, analysis of the data evidenced significantly low plasma levels for vitamins C and E, β-carotene, PSH, PON and Se when compared to the reference intervals. By contrast, concentrations of GPx, tROOH, Cu, CRP and IL-6, as well as Cu/Zn ratio were higher than the reference interval. All other OS biomarkers were unaffected in TAD patients. Supplementary Figure S1 shows that 66.6% patients had plasma values for vitamin C, PSH and Se below the low reference value (LRV) while 99% and 88% patients had higher levels of tROOH and Cu/Zn ratio, respectively, than the upper reference value (URV).

Significant correlations were observed between OS biomarkers (Table 3). The Cu/Zn ratio positively correlated with Cu, CRP, tROOH and IL-6 levels. Positive correlations were also observed between CRP and tROOH or IL-6. By contrast, CRP negatively correlated with Se and vitamin E and in the same way, IL-6 was also significantly negatively correlated with vitamin E, Se and PON.

### 3.3. Effect of Risk Factors on OSS

Smoking habits resulted in a vitamin C median value largely below the LRV and significantly lower compared to non-smokers (Table 4). However, the vitamin C median value in non-smokers only exceeded the LRV by 0.29 µg/mL. PON concentration was significantly lower in smokers compared to non-smokers and CRP levels were 8.64-fold higher in smokers than in non-smokers. The presence of hypertension led to median values of Cu, Cu/Zn and tROOH that were significantly higher than in the absence of this TAD risk factor. However, the median values in non-hypertensive patients were in the upper range of the reference interval (for Cu) or above the reference interval (for Cu/Zn and tROOH).

## 4. Discussion

The systemic OSS in TAD has been poorly investigated. In a recent study on 36 thoracic aortic aneurysm (TAA) patients, Irace et al. [22] evidenced a significant increase in serum hydrogen peroxide as a marker of ROS production when compared to a control group of 23 patients undergoing an aortic valve surgery. To the best of our knowledge, the systemic OSS determination in TAD patients in the early period following the diagnosis using a large number of OS biomarkers [23,33] has never been reported before our study.

### 4.1. Antioxidants

The low median value of vitamin C observed in TAD patients corresponds to the definition of a hypovitaminosis C (≤6 µg/mL) [34] known to be associated with a higher risk of developing cardiovascular events as reported by Gey [32]. The same author also concluded that the ideal vitamin C/vitamin E ratio, offering a maximal cardio-protective effect, must be higher than 1.3–1.5 when the concentrations of both vitamins are expressed in µM [32]. In our study, this ratio was lower (0.69) than the reference interval, although not significant.

Even if the median concentration of plasma β-carotene (0.15 µg/mL) was in the normal range (0.06–0.68 µg/mL), such a value is not to be considered as being an optimal one. In fact, Gey reported that β-carotene levels < 0.22 mg/L were associated with an increased risk of developing cardiovascular diseases [32].

The PSH pool was highly impacted downwards in our TAD patients. The PSH pool consists of ~70% of the single thiols of human serum albumin (HSA-SH), whose oxidation has been shown to occur in human diseases associated with increased oxidative stress [35].

GPx is an antioxidant enzyme requiring glutathione (GSH) and selenium as co-factors to reduce tROOH [36]. Even if the GPx median concentration was above the URV (Table 2), it is probably not sufficient given the elevated concentration of tROOH and the low levels of Se. Elevated GPx levels in the blood are in contradiction with observations made on aorta segments, in which a significant decrease in GPx activity was determined in patients with different aortopathies [15,18].

Linked to high-density lipoprotein (HDL), the main role of PON is to protect LDL from oxidative stress by hydrolyzing oxidized phospholipids and thus preventing the formation of atherogenic ox-LDL molecules [37]. Furthermore, PON also suppresses the differentiation of monocytes into macrophages in the subendothelial space, thereby limiting the process of foam cell formation and thereby reducing the formation of atherosclerotic plaques. Therefore, a reduced PON activity could be also associated with an increased risk of cardiovascular diseases [38]. Even if in the normal range, the serum PON activity measured in both type A and B patients was close to the LRV (Table 2).

As shown in Supplementary Table S1, a similar decrease in antioxidant concentrations was observed in both type A and B groups, except for vitamin C. In fact, the median concentration (2.58 µg/mL) in type A patients was significantly lower than the reference interval, while for type B patients the median value (6.54 µg/mL) was at the lower limit of the reference interval. Such a difference could be due to the major cardiac surgery that the type A TAD patients underwent, which involves a cardiopulmonary bypass (CPB) procedure.

In a recent paper, Hill et al. [39] reported that perioperative vitamin C concentrations after such surgery may drop to values below 3 µg/mL up to 24 h after surgery. Rodemesiter et al. [40] also described that there was no recovery in plasma vitamin C concentrations until discharge of the patient 1 week post-surgery. In our study, the blood sample was drawn 14.5 days (median) and 15.5 days (median) after the surgery and diagnosis, respectively. No information about the vitamin C status is available for such a long period. In cardiac surgery, papers reported the use of a single dose of 2 g vitamin C through an intravenous route prior to surgery or a supplementation with 1 g to 10 g of vitamin C per day in the postoperative phase [41]. All the observations presented in our work allow us to potentially ask to what extent a supplementation of antioxidants, and more particularly vitamin C, would be useful in TAD patients after diagnosis.

### 4.2. Trace Elements

It is well known that copper in excess exhibits pro-oxidant activities by inducing free radical formation (Fenton reaction), resulting in increased lipid peroxidation [42]. By contrast, on one hand, zinc is known to exhibit antioxidant properties as the main co-factor of superoxide dismutase (SOD) and, on the other hand, it acts as an inhibitor of free radical reactions induced by copper [43]. Therefore, the copper/zinc ratio is considered an excellent indicator of OS. The higher the Cu/Zn ratio, the higher the oxidative damage to lipids, as evidenced by elevated values of tROOH (Table 2) [44]. In 89% of patients, we found high Cu/Zn ratios (Supplementary Figure S1), which were significantly and positively correlated with tROOH levels (0.65, *p* = 0.0032) (Table 3). This is in accordance with observations described in other clinical situations associated with increased OS [45].

In 78% TAD patients, we observed a Se deficiency with levels being largely below the cutoff value of 73 µg/mL (Table 2 and Supplementary Figure S1). Selenium is an essential micronutrient and plays a crucial role in immune and antioxidant responses, regulation of inflammation and thyroid hormone metabolism [46].

### 4.3. Biomarkers of Lipid Peroxidation

Lipid peroxidation is a well-established molecular mechanism that plays a key role in the development of atherosclerosis, diabetes, cardiovascular diseases and chronic inflammation [47]. One of the major findings of our paper was that 17/18 TAD patients exhibited tROOH levels significantly higher (up to 3.3 fold) than the URV of 432 µM (Table 2 and Supplement Figure S1). By contrast, ox-LDL concentrations, considered as another index of lipid peroxidation, surprisingly remained within the norms in all TAD patients. Recently it has been suggested that the use of statins may be associated with improved outcomes in patients with thoracic aortic aneurysms [48]. A recent systematic review and meta-analysis performed by Jamialahmadi et al. [49] concluded that statins were able to inhibit the formation of ox-LDL. Such an association was, however, not determined in our pilot study since only 4 out 18 patients were on statin therapy. A similar increase (over the upper value of the reference interval) for tROOH, but not for ox-LDL, was observed in both type A and B TAD patients (Supplementary Table S1).

### 4.4. Inflammatory Markers

In agreement with Ye et al. [50], we found high plasma values for both CRP (>5 mg/L) and IL-6 (>1 pg/mL) in all TAD patients, indicating the presence of a severe acute or chronic inflammatory process. Inflammatory processes are known to be closely related to OS, with one process being easily induced by the other [51]. In our study, such a synergy is suggested by the highly negative correlation between inflammation and antioxidants (Table 3). In particular, CRP negatively correlated with vitamin E and Se, while IL-6 with vitamin E, Se and PON [52]. In both TAD groups, inflammatory biomarker levels were largely higher and to the same extent for both groups than the reference interval (Supplementary Table S1).

### 4.5. OSS in Genetically Triggered TAD

In a recent systematic review, Portelli et al. [12] explored the contribution of OS to the pathogenesis of genetically triggered TAA in aorta segments but not in blood samples. The authors showed the occurrence of OS in genetically triggered TAA, but were not able to explain the precise contribution of ROS to the pathogenesis of the disease. Due to our small sample size, we did not compare the systemic OSS of the group with genetically triggered TAA to patients who tested negative in the genetic analysis.

### 4.6. Limitations of the Study

The drawbacks of our pilot study include the small sample size and the single time-point measurement of systemic OSS parameters performed after the diagnosis of TAD (median 15.5 days). Therefore, the potential variation of these parameters over time was not evaluated. Moreover, many confounding factors might affect OSS, such as pre-existing cardiovascular risk factors (overweight, hypertension, diabetes, smoking, etc.), occurrence of TAD, open surgery or TEVAR, and intensive care unit or hospital stay, all of which were not analyzed in our study.

The plasma concentration of vitamin C (3.23 µg/mL) was largely lower than those reported in hypertensive (7.4 µg/mL) or diabetic (7.29 µg/mL) patients [53,54]. Moreover, the smokers’ plasma vitamin C concentration (9.7 µg/mL) reported by Giraud et al. is still within our reference interval [55]; therefore, smoking habits cannot explain the low vitamin C values of our TAD patients. The present study highlighted that the median plasma vitamin C concentration (3.23 µg/mL) in TAD patients was largely below those associated with cardiovascular risks. However, the surgery procedure required in type A TAD patients must be taken into consideration, since the vitamin C analysis was performed after surgery [39].

We measured blood plasma levels of GPx and PON which belong to the antioxidant enzyme family; however, in further studies SOD should be included as well. Having the values for the three enzymes should allow a better comparison of the blood data with the data described in aortic segments. Moreover, it would be interesting in future studies to perform the OSS evaluation at the time of admission of the patient into the intensive care unit, rather than 15 days post-diagnosis and eventually follow the evolution of the disease.

Given that our study was a pilot study, the current findings warranty further research.

## 5. Conclusions

For more than 30 years, our hospital center has acquired an extensive expertise in the screening, diagnosis, surgical treatment and therapy of different aortopathies. Recently, we initiated the local LIDIA study on TAD patients in order to better characterize its incidence and prevalence in Liège Province, Belgium. Based on recent literature, there is increasing evidence that oxidative stress could be involved in the development of this disease. In fact, it has been observed that aortic segments, drawn during urgent surgical procedures in type A TAD patients, show altered levels of antioxidant enzymatic activities [15,16,17,18,19,20].

As part of the LIDIA study, this paper evaluated the systemic OSS in TAD patients after TAD diagnosis by measuring a large panel of biomarkers routinely used in our hospital center for many years. The present pilot study on TAD patients clearly revealed a significant decrease in the concentration of low-molecular-weight antioxidants, specifically vitamin C, thiol proteins, β-carotene, and increased lipid peroxidation in type A TAD patients requiring urgent surgical procedures after their diagnosis. Interestingly, we noted a similar altered OSS in type B TAD patients not requiring urgent surgery, which is an event that could potentially have an influence on the OSS. Such results in both groups of TAD patients, which need to be confirmed on a larger scale, should encourage clinicians to integrate some OS analyses into routine biological assessments with, of course, great precautions in the pre-analytical treatment of blood samples.

## Figures and Tables

**Table 1:** Demographic, biometric and medical characteristics of TAD patients. Age and BMI were expressed as median (IQR). BMI, Body Mass Index; HT, hypertension; CAD, Coronary Artery Disease; PAD, Peripheral Arterial Disease; RI, Renal Insufficiency; COPD, Chronic Obstructive Pulmonary Disease; NSAID, Non-Steroidal Anti-Inflammatory Drug.

Variable	Total, N = 18 (%)
Age (years)	62 (55–68)
Gender (M/W)	10/8 (56/44)
BMI (kg/m^2^)	27.0 (23.7–28.4)
Smoking Status	
Never	4 (22)
Former	5 (28)
Current	9 (50)
Diabetes	3 (17)
HT	14 (78)
Dyslipidemia	7 (39)
CAD	1 (5)
PAD	2 (11)
RI	6 (33)
COPD	3 (17)
Other Aneurysms	2 (11)
NSAID	7 (39)
β-blockers	4 (22)

**Table 2:** Comparison of OS biomarkers in TAD patients (N = 18) with reference intervals. Statistical significance was determined using the binomial sign test (see Statistical analysis paragraph in Section 2 for details). K is the number of TAD patients whose values fall below (L) or above (H) the middle of the reference interval.

Parameters	Reference Interval	Median (IQR) (N = 18)	K	*p*-Value
Vitamin C (µg/mL)	6.21–18.00	3.23 (1.84–7.35)	16 (L)	<0.002
Vitamin E as α-tocopherol (µg/mL)	8.60–19.24	12.03 (9.91–13.22)	16 (L)	<0.002
Vitamin E as γ-tocopherol (µg/mL)	0.39–2.42	<0.39	16 (L)	<0.002
Vitamin C (µM)/Vitamin E (µM)	1.3–1.5 *	0.69 (0.5–1.80)	10 (L)	1
β-carotene (µg/mL)	0.06–0.68	0.15 (0.08–0.31)	18 (L)	<0.0001
PSH (µM)	314–516	246 (216–330)	17 (L)	<0.001
GPx (IU/g Hb)	20–58	61 (55.5–84)	18 (H)	<0.0001
PON (IU/L)	39–408	62.7 (42.4–134.6)	17 (L)	<0.001
Se (µg/L)	73–110	64.8 (53.8–83.1)	15 (L)	0.008
Cu (mg/L)	0.70–1.1	1.36 (1.14–1.58)	17 (H)	<0.001
Zn (mg/L)	0.70–1.20	0.87 (0.67–0.97)	9 (L)	1
Cu/Zn	1–1.17	1.63 (1.44–2.00)	17 (H)	<0.001
tROOH (µM)	0–432	1439 (785–1901)	18 (H)	<0.0001
Ox-LDL (ng/mL)	28–70	36.5 (31–49.75)	4 (H)	1
CRP (mg/L)	0–5	67.6 (12.05–124.6)	18 (H)	<0.0001
IL-6 (pg/mL)	0–1 **	14.07 (7.49–26.88)	18 (H)	<0.0001

* Normal value proposed by the High Sensitivity Human IL-6 Quantikine Elisa kit purchased from R&D, Abingdon, UK. ** Reference interval proposed by Gey et al. [32].

**Table 3:** Correlation between all investigated OS biomarkers observed in TAD patients (N = 18). Correlation between biological factors was established using the Spearman test for nonparametric data.

Association		Correlation	*p*-Value
Cu/Zn	Copper	0.58	0.010
Cu/Zn	CRP	0.90	<0.0001
Cu/Zn	tROOH	0.65	0.0032
Cu/Zn	IL-6	0.66	0.002
CRP	tROOH	0.59	0.009
CRP	IL-6	0.75	0.0003
CRP	Selenium	−0.47	0.049
CRP	Vitamin E	−0.48	0.04
IL-6	Vitamin E	−0.54	0.026
IL-6	Selenium	−0.53	0.022
IL-6	PON	−0.49	0.037

**Table 4:** Influence of TAD risk factors such as smoking and hypertension on several markers of OS. Values for the two groups, smokers versus non-smokers, are expressed as median (IQR).

Parameters	Reference Interval	Smoking Habits		*p*-Value
		No (N = 9)	Yes (N = 9)	
Vitamin C (µg/mL)	6.21–18.00	6.5 (2.6–9.3)	2.6 (1.5–3.5)	0.05
PON (IU/L)	39.5–408.2	132.6 (72.6–141)	43 (32–54)	0.0008
PSH (µM)	314–516	283 (246–359)	227 (212–246)	0.070
CRP (mg/L)	0–5	12.8 (8.4–56.3)	110.6 (87.3–159)	0.011
		**Hypertension**		
		**No (N = 4)**	**Yes (N = 14)**	
Cu (mg/L)	0.70–1.1	1.00 (0.91–1.10)	1.47 (1.27–1.67)	0.011
Cu/Zn	1–1.17	1.28 (0.99–1.50)	1.78 (1.60–2.05)	0.025
tROOH (µM)	0–432	689 (514–828)	1630 (1353–2023)	0.012

## Data Availability

The data are contained within the article and Supplementary Materials.

## Supplementary Materials

The following supporting information can be downloaded at: https://www.mdpi.com/article/10.3390/antiox12051106/s1. Figure S1: Individual plasma values of vitamin C, thiol proteins, selenium, lipid peroxides and Cu/Zn ratio. Black circles: TAD A patients, grey circles: TAD B patients; Table S1: Comparison of OS biomarkers between type A and B TAD patients.
