# Supplementary material for: Alteration of Blood Oxidative Stress Status in Patients with Thoracic Aortic Dissection: A Pilot Study
Source: Antioxidants (Basel). 2023 May 16;12(5):1106. doi: 10.3390/antiox12051106 (PMC10215099; doi:10.3390/antiox12051106)
Supplement: Supplementary file 1 [file antioxidants-12-01106-s001.zip › antioxidants-2259357-supplementary.pdf]

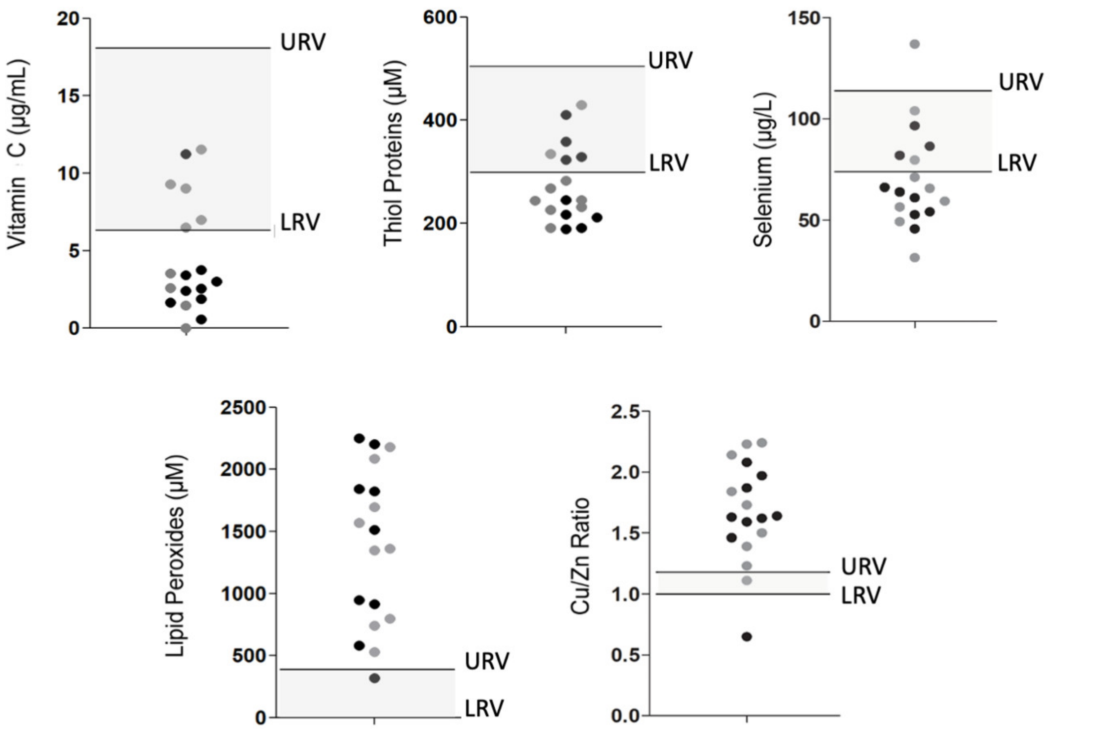

Figure S1. Individual blood values (N=18) of vitamin C, PSH, Se, Lipid Peroxides and Cu/Zn ratio. Black dots: type A TAD patients; gray dots: type B TAD patients.
